# Temporal and Individual Variation in Offspring Provisioning by Tree Swallows: A New Method of Automated Nest Attendance Monitoring

**DOI:** 10.1371/journal.pone.0004111

**Published:** 2009-01-01

**Authors:** Alexandra P. Rose

**Affiliations:** Department of Ecology and Evolutionary Biology, University of California Santa Cruz, Santa Cruz, California, United States of America; University of Bristol, United Kingdom

## Abstract

Studies of the ecology and evolution of avian nesting behavior have been limited by the difficulty and expense of sampling nest attendance behavior across entire days or throughout a substantial portion of the nestling period. Direct observation of nesting birds using human observers and most automated devices requires sub-sampling of the nestling period, which does not allow for the quantification of the duration of chick-feeding by parents within a day, and may also inadequately capture temporal variation in the rate at which chicks are fed. Here I describe an inexpensive device, the Automated Perch Recorder (APR) system, which collects accurate, long-term data on hourly rates of nest visitation, the duration of a pair's workday, and the total number of visits the pair makes to their nest across the entire period for which it is deployed. I also describe methods for verifying the accuracy of the system in the field, and several examples of how these data can be used to explore the causes of variation in and tradeoffs between the rate at which birds feed their chicks and the total length of time birds spend feeding chicks in a day.

## Introduction

Studies of avian nesting behavior have played a central role in the development of several areas of evolution and ecology, including life history evolution [e.g. 1], [2], parental investment [e.g. 3], and mating system evolution [e.g. 4]. Empirical studies in all of these areas often rely on an ability to accurately quantify the amount of time that individual birds or pairs invest in incubating and brooding chicks, or the rate at which parents feed their offspring. As a result, studies rely on lengthy, labor-intensive observation of birds simply coming and going from their nests in order to explore nest building behavior, the physiology of incubation, chick feeding behavior, sex ratios of parental care, time budgets of breeding birds, and comparative work on parental investment and life histories [e.g. 3], [5], [6], [7]. Not only is this work time-consuming, it requires tremendous attention to detail and considerable observer stamina, as many birds have evolved elaborate mechanisms to avoid being seen as they enter and exit their nests. Observation bouts may be lengthy and must be performed at all hours of the day and in harsh physical conditions. Ecologists are often concerned that direct observation of birds at their nests may also be disruptive and can decrease nesting success or even lead to abandonment [8]. Because direct observation necessarily sub-samples nesting behavior, it may therefore add considerable amounts of sampling variance to estimates of total time devoted to different behaviors. Additionally, it potentially limits the ability of researchers to obtain extensive and thorough data on temporal and spatial variability in nesting behaviors. As such, reliance on direct observations has limited our understanding of the importance of the total amount of time birds spend provisioning chicks and how the duration of provisioning behaviors trades off with other aspects of chick rearing. In short, the need to collect extensive individual data in order to quantify variation in nesting behaviors is a critical limitation in many evolutionary and ecological studies.

To circumvent the restrictions on accuracy and sample size that are imposed by direct observation of breeding birds, researchers have developed many different ways of automating the collection of nest attendance data including: video taping [e.g. 9]; time lapse photography or motion triggered cameras [10]; use of data logging thermometers such as an Onset Hobo® Logger and Dallas Semiconductor Thermocron iButtons [11], [12]; weigh bridges or electronic balances [13], [14]; light sensors [15]; transponders attached to leg bands [16], and mechanical visit counters [17]. All of these methods have their advantages and limitations and may be beneficial for particular studies — see [8] for a thorough discussion of these various techniques and their applications – but none to date have affordably extended the range of observations to include all, or even the majority, of the chick rearing period for a large number of breeding pairs.

My goal was to design a fully-automated method for measuring nest attendance throughout entire 24 hour periods and over the entire chick rearing period in order to explore temporal variation in chick feeding rates driven by weather and other variables. This device was designed specifically for cavity nesting species but is potentially adaptable to cup nesters as well. This Automatic Perch Recorder (APR) system is ideal for remote field conditions; it is relatively inexpensive (∼US$100), waterproof, rugged, self-powered, and can continuously record data on ambient temperature and parental nest attendance for more than 30 days—enough to encompass the incubation and nestling period of all cup nesting and cavity nesting passerines. The APR records the time and temperature at which birds become active in the morning, either commencing incubation bouts or feeding and brooding chicks. The APR is an especially powerful tool because it records every visit made to the nest by both members of a pair for as long as it is deployed, it tracks the ambient temperature throughout the day for that nest, and records the time at which birds become inactive for the night. This allows for the collection of accurate, long-term data on hourly rates of nest visitation, the duration of a pair's workday, and the total number of visits the pair makes to their nests—unique data that are prohibitively labor-intensive to collect using direct human observations or most existing automated devices. While the APR does not distinguish between the sexes in species where both members of the pair care for chicks, limited, direct observations can be made to estimate the ratio of visits made by males or females.

After describing the APR, I discuss two findings relevant to the goal of exploring temporal variability in chick feeding rates: what tradeoffs individual pairs make in the rate at which they feed their chicks versus the duration of their workday, and how chick-feeding behavior is affected by several weather variables. I also describe methods for verifying the accuracy of the APR using direct observation or video recordings. This allows one to correct for differences in how the APR is mounted at the nest entrance as well as for variation in how different species or individuals might use the perch that triggers the recording of a visit.

## Materials and Methods

### Study Area and Species

This study was conducted on private property adjacent to Elkhorn Slough National Estuarine Reserve on Monterey Bay, California. A population of over 80 pairs of tree swallows (*Tachycineta bicolor* (Vieillot, 1808)) breeds in nest boxes around brackish and freshwater ponds on this property. APRs were deployed on 8 different nest boxes in 2006 and 18 different nest boxes during the breeding season of 2007. In both seasons, I used APRs to record data from the date of hatching or earlier to when the chicks were at least 15 days old (∼356 days of 24 hour observations).

### Construction of Automatic Perch Recorders

**Figure 1:**
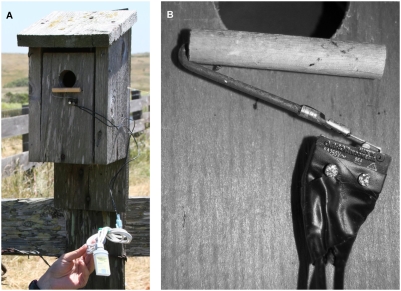
Nest box with Automatic Perch Recorder attached. The apparatus consists of a dowel-perch attached to a microswitch mounted at the cavity entrance (close-up in panel B), which is in turn connected to an event recording data logger—visible in bottom right of panel A.

An APR consists of two primary components—a weight-triggered electric switch attached to a perch and an event recording data logger (Figure 1). I used a hinge lever subminiature basic switch (Omron Electronics part #SS-5GL, US$1.59) that requires 50 g of contact force to close the circuit. Since the perch is necessarily cantilevered on the switch lever and because the birds land with some force on the perch as they arrive at the nest, this amount of contact force is reliably generated by the weight of the perch (∼10 g) and the tree swallows, for which the mean weight of an adult in this population is 18.9 g (unpubl. data). Perches are made of a 1 cm diameter dowel cut into 5 cm lengths and drilled so that a 8 cm piece of 18 gauge galvanized multi-purpose wire can be inserted into its center. The wire is secured inside the dowel with glue, and its protruding end is bent into a U shape. The end of the wire not attached to the dowel is soldered to a 22-18 gauge female vinyl-insulated barrel disconnect that is crimped around the wire. The female end of the connector is then soldered or attached with metal epoxy to the lever arm of the switch. The data logger component of the APR is a Hobo® Pendant Event Data Logger (Onset part #UA-003-64, US$89) programmed to record the time of every switch closure as well as to take a temperature reading every 10 minutes throughout its deployment. The leads on the data logger may be attached directly to the switch or connected with 22-18 gauge fully-insulated disconnects to leads attached to the switch. The latter arrangement allows loggers to be easily moved among nests and removed for uploading data and redeployment. APRs were mounted on nest boxes so that the perch covered approximately 1 cm of the entrance of the box (Figure 1) and they were attached using nails driven through the mounting holes of the switch. APRs were mounted on nest boxes during or after clutch completion, during incubation, and in some cases after hatching.

Practical design features to make the APR accurate and durable include: A) the perch must be positioned high enough so that it will reliably be triggered as a parent enters the nest, but low enough that it does not block the entrance and become triggered as birds are leaving the nest; B) the weight and shape of the perch, wire, and switch lever must be correctly gauged so that a landing bird will reliably depress the switch and that it will reliably rebound; C) the switch must be attached securely enough so that it does not loosen with time, but care needs to be taken to ensure that the switch and perch do not touch (and hence encounter friction with) the wall of the nest box, and D) while the system described above is completely waterproof, the insulated wire leads that connect to the switch should be wrapped in electrical tape or otherwise sealed so that they don't corrode and lose conductivity. Other switch sizes or types would be appropriate for other species, and any event recorder with enough memory to record large numbers of events could be used to build a similar system. Tree swallows tend to be relatively tolerant of disturbance at the nest and did not respond adversely to having the APR mounted on their nest box at any stage of nesting—during nest building, egg laying, incubation or after hatching. Other species may require that the APR be mounted prior to egg laying (limited to cavity nesting birds) or after clutch completion or hatching depending on the sensitivity of the focal species.

A key advantage of this system is that with a laptop computer, data may be downloaded directly from the APRs without removing them from the nest box. Since downloading even a month's worth of data takes approximately 1 minute, this is easily done without greatly disturbing the nesting pair. This capability allows for practically infinite deployment durations. Alternatively, the data logger may be disconnected from the leads attached to the switch and downloaded away from the nest or at a later time. To download the data from the data logger, I used the Hobo® Optic USB Base Station (Onset part #BASE-U-1, US$59) which connects the data loggers to a computer via a USB port. Data may be read off the loggers with one of several versions of HOBOware® and exported to Excel or other spreadsheet program for analysis.

### Validation of APR data

One problem with the use of a perch activated event recorder is the possibility that birds might occasionally fail to trigger the logger as they enter the nest and they might also trigger the logger as they leave the nest. To quantify these potential problems and understand how best to correct for them, I simultaneously obtained video data and APR data for the same nesting pairs. I used the video observations to validate the APRs by comparing the number and timing of parental visits (“events”) recorded by the APRs to the number and timing of visits observed on the videos. Specifically, in 2006 I filmed each of the 5 nests with APRs when chicks were between 6 and 12 days old for 6–8 hours using Sony 8 mm Camcorders. I sub-sampled the more than 50 hours of video by using 15 minutes of every hour of film for all nests, resulting in 33 separate 15 minute intervals of analyzed visitation data. I recorded the time when birds arrived at their nests on the video and then compared those times to the visits logged by the APRs. I then categorized each visit logged by the APR as either a “real” or “false” event depending on whether it corresponded with a parental visit recorded on the video.

## Results

### Testing the functionality and accuracy of the Automatic Perch Recorder

In 2007, birds (N = 18) rapidly returned to their nests and resumed brooding or feeding after APRs were mounted (mean = 27, range = 14 to 42 minutes). Out of approximately 75 nests on which APRs have been mounted over the course of two breeding seasons, in two different locations, the presence of an APR has never caused a pair to abandon their nest.

When adult tree swallows are actively feeding chicks, they tend to arrive at the nest, deliver food to the young, and then depart quickly. The video recordings allowed me to see that “false” events were mainly caused by birds stepping on the perch of the APR as they left the nest after feeding. Because birds usually spend only a brief amount of time in the nest cavity when they are feeding chicks, I suspected that I could distinguish the “false” events from the “real” events –recorded as the birds arrive at the nest entrance – by the time interval between one event and the preceding event. To evaluate this, I determined the interval in seconds between every pair of consecutive events recorded by both the video recordings and APRs. I then used a simple Matlab® program (*available from author*) to examine the accuracy of using different cutoff intervals to eliminate “false” events. For each cutoff interval, I classified an event as real if the time between it and the previous event was greater than the cutoff and false if it was less than the cutoff: the proportion of all events that were misclassified then gives a measure of the best cutoff to use. I quantified accuracy as the absolute difference between the number of real events observed by video during a 15 minute observation interval and the number of events recorded by the APR and scored as real using a given cutoff. I expressed these differences as proportions of the number of video-recorded real events and took the mean over the 33 observation periods as an overall gauge of accuracy.

**Figure 2:**
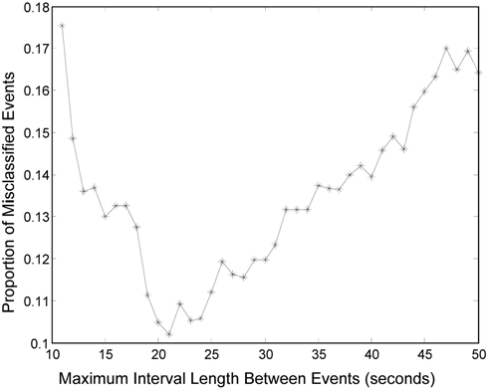
Proportion of all events misclassified using different interval length cutoffs to classify events as “real” or “false”. All events with intervals less than a given cutoff were classified as false, and those with intervals greater than the cutoff were classified as real. Video recordings were then used to determine what proportion of events were misclassified. When visits with an interval less than or equal to 21 seconds are excluded, the error rate is lowest at 10.2%.

Using a cutoff of 21 seconds results in an accuracy of 89.8% in terms of the proper classification of individual events as either “real” or “false”. Using cutoff intervals longer or shorter than 21 seconds resulted in much larger error rates—using intervals shorter than 21 seconds includes false events, and using intervals longer than 21 seconds means that an increasing number of real events are excluded (Figure 2). At the level of individual events, this cutoff leads to correct classification of 86.5% of events with preceding intervals of greater than 21 seconds, which comprise 67.8% of all events. However, of the 32.2% of remaining events that had intervals between them which were shorter than 21 seconds, only 67.5% were correctly classified as false. This means that the remaining events were real events which were deemed false because they were separated by an interval of less than 21 seconds. However, over the entire sample of data, using the 21 second interval as a filter to exclude false events from the logger data actually makes the APRs far more accurate than 89.8%, since misclassified events will cancel one another out in terms of the total count. As a result, using the 21 second threshold, the filtered APR data counted 246 events, while the video count was 237, resulting in 96.3% accuracy overall.

The best cutoff interval to use may vary depending on the nesting stage and/or age of the chicks in the nest, since the average duration of visits to the nest will change across the nesting period. To account for this fact, and for the inevitable variation in the exact construction materials and mounting methods used for the APRs, it is necessary for anyone who uses the APR to use the validation procedure to determine the best cutoff interval for their specific situation. By doing so, it will be possible to minimize the error rates for the particular nesting stages of interest.

Modifying the way the perches are mounted on the nest boxes might be one way to reduce the error rate even further. For example, perches could be mounted on the interior of the nest entrance so that birds hit the perch when entering and exiting. Eliminating half the events logged would then give an accurate count of visits. Such an arrangement, or the simultaneous deployment of two APRs on either side of the nest entrance, could also provide the possibility of using APRs to examine the length of incubating and brooding bouts, as birds would trigger one APR as they arrived at the nest and the other when they departed.

### Demonstrating the utility of the Automatic Perch Recorder

**Figure 3:**
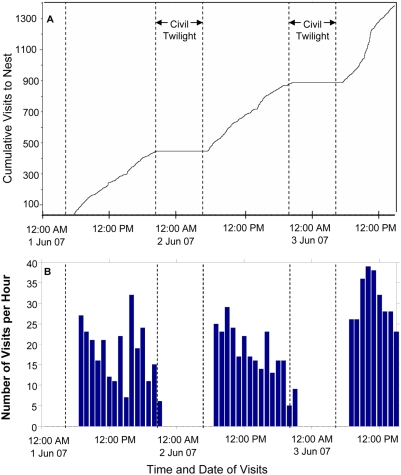
Automatic Perch Recorders count a “visit” each time parents arrive at their nest. A) a two and a half day span of time and the cumulative number of feeding visits made to the nest during that time with dashed lines showing the beginning and end of civil twilight; and B) the hourly rate of parental feeding visits to the nest over the same time period.

Data from the APRs give a high resolution picture of nest visitation activity. During incubation and the nestling period, the APRs show the time at which brooding females leave the nest in the morning, the number and timing of the intervals that they spend incubating or brooding, away from the nest, or foraging for chicks, and the time of the final return to the nest in the evening (Figure 3). Perhaps most uniquely, this method allows the simultaneous quantification of these nest attendance behaviors for multiple pairs across many days of activity. Using data from APRs on the rate and duration of chick-feeding for multiple pairs of birds across their nestling periods, which are for the most part overlapping, I examined: a) how weather conditions affect chick-feeding, and b) how birds balance the hourly rate at which they feed their chicks with the total length of their workday.

**Figure 4:**
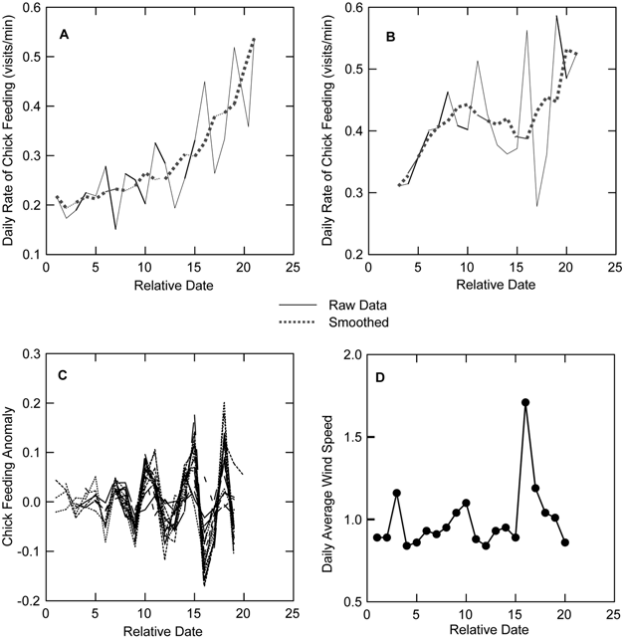
The mean daily rate of chick feeding is highly variable across time within and between nests and shows a response to wind speed. Panels A and B show the rate of chick-feeding (solid line) across time for two different nests, and the moving 5 point average of the chick-feeding rate (dashed line). Panel C shows the chick-feeding anomaly–the difference between a pair's daily chick-feeding rates and the moving 5 point averages–for all pairs across the nesting period, and it demonstrates how daily rates of chick-feeding change synchronously across pairs. Panel D shows the average wind speed for each day of the chick-rearing period. Relative date is the number of days since the hatch date of the first nest in the population.

Perhaps the most striking pattern documented by the APRs is the highly synchronous, cyclical variation in feeding rates across all 18 pairs followed in 2007 (Figure 4c; two-way ANOVA of rate on pair and date combinations without missing values, using Greenhouse-Geisser epsilon correction for repeated measures [per 18]: date *F*_3.36, 47_ = 29.69, *P*<0.0001; pair *F*_14,182_ = 27.23, *P*<0.0001). The work of McCarty [9], who used video cameras to film the interior of nest boxes and showed that parent tree swallows feed nestlings on 95–98% of visits to the nest, allows me to use visit data from the APRs as a proxy for chick-feeding behavior. I subsequently created a variable to separate the variation of interest from the general increase in feeding rate with chick age that is well known in many passerine species [e.g. 19]. This variable, which I will call the “chick feeding anomaly”, is equal to the difference between each pair's daily chick-feeding rate and a 5 point moving average of those rates across the chick rearing period (e.g. Figure 4a and 4b). I then used multiple regression analyses to assess the impact of several weather variables on the chick feeding anomaly. Regressors were average wind speed, speed of fastest gust in a day, mean, minimum and maximum air temperature, and mean, minimum, and maximum relative humidity. I ran 14 models with different subsets of these variables, and used AIC criteria values and AIC weights to gauge support for different models and explanatory factors. These analyses showed that the model with average wind speed and average temperature had the highest support (AIC weight = 0.287) but that a model with wind speed alone was nearly as well supported (AIC weight = 0.236, Table 1). Furthermore, summed AIC weights across models show strong support for wind speed as an explanatory variable (summed AIC weight = 0.966), but not for any other factor, including mean temperature (summed AIC weight = 0.596). Not surprisingly, increased wind speed reduces the rate at which birds return to their nests (for the best fit model, wind regression coefficient = −0.305, *F*_6,13_ = 2.145, *P* = 0.117, *r^2^_adj_* = 0.266). A one-way regression of the effect of average wind speed on the chick feeding anomaly showed that average wind speed explains 26.7% of the variation in the chick-feeding anomaly (*F*_1,18_ = 6.54, *P* = 0.020, *r^2^* = 0.267). This effect may be due directly to the difficulty of finding aerial insects on windy days in combination with the increased difficulty of flying between the nest and foraging areas in windy conditions. Further exploration of the effect of wind on the feeding rates of the birds shows that the birds' behavior is not only negatively affected by the wind they experience on a given day, but it is even more strongly positively affected by the wind conditions they experienced the previous day. A multiple regression including the effects on the chick feeding anomaly of the log-transformed average wind speed of the present day, log-transformed wind speed the previous day, and an interaction term for present and prior wind speed, shows highly significant positive effects of both previous day's wind speed (*F*_3,197_ = 16.21, *P*<0.001) and the interaction term (*P* = 0.019). The regression data for each factor is given in Table 2. To further explore the patterns of feeding rate variation and wind speed, I also conducted a time series analysis on each pair's feeding rate anomaly and on the analogous anomalies of wind speed from a 5 point moving average of these daily rates. The autocorrelation values for lags of 1, 2, and 3 days for wind speed (−0.278, −0.332, and 0.231, respectively) show a close correspondence with autocorrelations of feeding rates (mean±SE) for the same lags (−0.232±0.043, −0.443±0.040, and 0.250±0.038, respectively). These results also suggest that the patterning of feeding rate is driven by wind speed or perhaps by some other, closely correlated, environmental variable.

**Table 1:** Variables included in each regression model and their AIC results.

Maximum Daily Wind Speed	Mean Daily Wind Speed	Mean Daily Humidity	Mean Daily Temperature	Maximum Daily Temperature	Minimum Daily Temperature	*Maximum Log Likelihood*	*AIC Criteria*	*AIC Weights*
x						43.155	−81.603	0.009
	x					46.392	−88.079	0.236
		x				43.155	−81.603	0.009
x		x				43.155	−78.809	0.002
	x	x				47.316	−87.132	0.147
	x	x	x			47.983	−85.300	0.059
	x	x		x		47.644	−84.622	0.042
	x	x			x	47.983	−85.300	0.059
	x		x			47.983	−88.466	0.287
	x			x		46.691	−85.882	0.079
	x				x	46.392	−85.285	0.058
		x	x			43.814	−80.128	0.004
		x		x		44.044	−80.588	0.006
		x			x	43.155	−78.809	0.002

**Table 2:** Effects of Present Day and Previous Day Wind Speed on the Chick Feeding Variable.

Factor or Interaction	Coefficient	Std. Error	t statistic	*p* value
Avg. Daily Wind Speed Present	−0.019	0.047	−0.411	0.681
Avg. Daily Wind Speed Previous	0.3	0.045	6.666	<0.001
Avg. Daily Wind Speed Present×Avg. Daily Wind Speed Previous	1.027	0.432	2.375	0.019
Constant	0.016	0.005	3.447	0.001
**R Squared**	0.198			
**Adjusted R Squared**	0.186			

**Figure 5:**
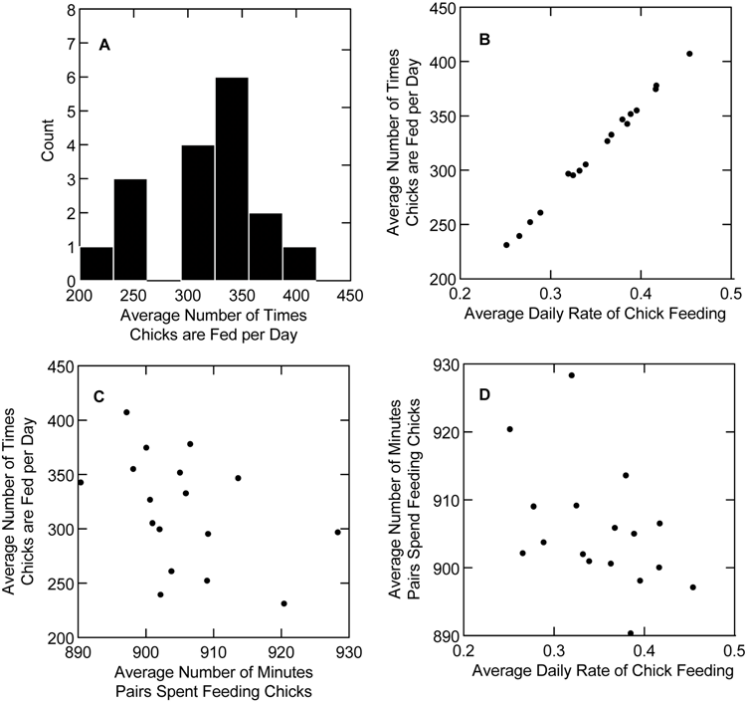
Variation in the number of times parents feed chicks per day and the relationships of feeding rate, duration of feeding, and number of times parents feed chicks. a) Histogram showing variation in the average number of times chicks were fed per day by 17 different pairs of birds. The mean number of times chicks were fed per day by all pairs is 317.4. b) The number of times chicks were fed each day was strongly predicted by the rate at which parents fed chicks. c) The number of times chicks are fed on average is negatively related to the average amount of time pairs spent feeding chicks in a day. d) There is also an inverse relationship between the number of minutes birds spent feeding their chicks and the rate at which they fed them.

In addition to showing synchronized fluctuations in chick-feeding rates across days, pairs also show substantial additional variation in the number of times chicks are fed per day, both within and between pairs (Figure 5a; mean±SD = 312.137±54.424). The APRs provide an easy way to evaluate whether this variation is due to differences in the duration of time that pairs spend feeding chicks each day (i.e. the length of their workday) or differences in rate of visits to the nest during the period of active feeding each day (mean duration (minutes)±SD = 896.902±8.819; mean rate (visits per minute)±SD = 0.348±0.058). My data show that, during the period when there is active foraging, visits occur virtually without pause, with an abrupt beginning and end to the foraging period (Figure 3). This makes it reasonable to break foraging behavior into two variables: rate and duration.

Since total the total number of times chicks are fed in a day is the product of the average chick-feeding rate and the total duration of the active chick-feeding period, their individual variances and their covariance must together explain all variation in visit number. Using the standard delta method [20] to decompose variance, variation across pairs in mean daily visit number (*V*), can be broken into the sum of the scaled variances and covariance of *R*, a pair's mean daily rate, and *D*, their mean daily duration: 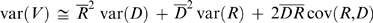, where 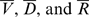 are the across-pair means of the three variables. The proportional contribution of each term in this sum thus indicates the contribution of each variance or covariance term to variation between pairs in mean daily visits.

I found that variation in the rate of visits explains nearly all the total observed variance in V (Figure 5). The main effect of variation in *R* is 2755.92, or ∼278 times the main contribution of variation in the length of the workday, *D* (9.93). Interestingly, the relationship of the duration of time that pairs spend feeding chicks each day is *negatively* related to the rate at which they feed—birds that work longer days tend to feed at a slower rate as compared to birds that work shorter days and the effect of this negative covariance 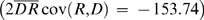 is actually a stronger determinant of var(*V*) than is variance in workday duration.

## Discussion

Automatic Perch Recorders are a powerful tool for exploring many different aspects of nest attendance behavior in a non-invasive way. Data from the APR can be uploaded and analyzed instantaneously after validating as opposed to many other ways of gathering similar data where human observations and video recordings need to be coded and transcribed. This capability reduces labor, error, and cost. APRs are able to gather a more accurate and detailed picture of nesting behavior not only because they are always “on”, but also because they eliminate the possibility of transcription errors. Also beneficial for remote field conditions is their extreme portability and the ability to deploy them and leave them unattended for weeks if desired.

The completeness of the picture of nest attendance given by the APRs along with their low cost affords opportunities for improved understanding of how birds translate parental effort into nesting success. Prior work on parental effort has involved extrapolating from short term data on nest visitation rates (i.e. the number of visits in a span of hours), often collected during a brief span of the nestling period, to estimate longer term rates of nest visitation (i.e. daily or weekly rates of visitation). Because APRs collect data on both short term rates, long term rates, and the duration of the active period, they provide the data needed to partition variance in parental effort and understand exactly how birds trade off short term energy expenditure with the amount of time they spend feeding chicks each day across the entire nesting period.

Because APRs allow for the simultaneous quantification of nest attendance behaviors for multiple pairs across many days of activity, they permit thorough exploration of both temporal and spatial variation in these behaviors. Studies of nesting behavior are often limited in their ability to sample across time and space by the need to achieve adequate sample size in terms of numbers of nests monitored. APRs help resolve this problem by reducing the number of observer hours needed to collect and transcribe nest attendance data. The APR system has particular potential for studies of cavity nesting birds where deploying great numbers of them across large geographic scales, especially at nest box arrays, would be logistically feasible and could elucidate questions regarding how temporally and spatially variable ecological factors such as weather, food resources, predation, daylight, and length of breeding season might effect the rate, duration, and trade-offs in nest attendance behaviors. Studies of this sort in conjunction with studies of demography, physiology, and nesting success have particular potential to help us understand the effects of trends in habitat modification and climate on avian populations.

My example analyses illustrate some of the advantages of APRs. The result that wind conditions limit the rate at which this population of aerial insectivores feeds their young is not surprising. However, the strength of the pattern of inhibition by wind demonstrates that weather can be a severely limiting factor for these birds and that the design of observational studies and experiments involving foraging success and chick growth must take into account the problem of wind. Interestingly, cool, wet days with low wind do not slow the rate of food delivery to the nest to the extent that a windy day with otherwise benign weather conditions does.

The strong positive effect of the previous day's average wind speed on the rate at which birds feed their chicks during the current day shows that these birds are making decisions regarding how to balance their chicks' needs for food with the most efficient use of their own energy for foraging. My analysis clearly shows that birds feed their chicks more rapidly on the current day if it had been windy the previous day. If it was windy the previous day and still windy on the current day, birds slightly increased their rate of feeding as compared to the previous day, but not as much as if it were currently a calm day. However, wind speed explains only approximately one third of the variation in the chick feeding anomaly, suggesting that the strikingly cyclic pattern of chick feeding rates across nests is also driven by one or more additional factors and may be evidence of complex social dynamics in the foraging behavior of this population. Since swallows are highly social birds that forage together in large groups it is not surprising that that the population would respond synchronously to environmental variability, however the strength of this pattern is especially remarkable considering that the birds in this sample were random with respect to age, quality, and the age and condition of their young.

The APR also provides the ability to indirectly explore mechanisms behind variation in other chick feeding behaviors. Different pairs of birds in this population show strong variation in the number of times each day that they feed their chicks. While the source of this variation appears to be differences in the rate at which birds feed their chicks, it would be interesting to determine whether the variability in rate is caused by differences in foraging success (i.e. the amount of food a bird is able to gather in a certain period of time), the number and condition of chicks in a nest, habitat variation and many other potential drivers. Regardless, the negative relationship between the rate at which birds feed their chicks and the duration of the day that they spend feeding chicks suggests that more successful pairs (those with higher overall visit numbers due to higher rates) are able to marginally reduce the duration of their workday due to their higher return rate. Understanding what drives the variability in the rate of chick feeding is essential to elucidating the mechanisms of how differences in individual quality translate into nesting success.

In addition to the limited examples presented here, there are many other potential ways to employ APR devices. The APR gives excellent data on the timing of fledging without being at all intrusive. This device also has the potential to further understanding of patterns of nest site visitation for cavity nesters during nest building, egg laying, and incubation. The APR can also be used to explore patterns of roosting behavior outside of the breeding season either by deploying them at nest boxes for species that are known to roost in the vicinity of their breeding cavity or in the cavities of other birds. I have also used the devices to understand when otherwise unobserved nest predation events occurred. Finally, with some modification, APRs may be adaptable to cup nesting birds with reasonable tolerance for foreign objects at their nests.
